# Physical fitness reference standards for Chinese children and adolescents

**DOI:** 10.1038/s41598-021-84634-7

**Published:** 2021-03-02

**Authors:** Feng Zhang, Cunjian Bi, Xiaojian Yin, Qi Chen, Yuqiang Li, Yuan Liu, Ting Zhang, Ming Li, Yi Sun, Xiaofang Yang

**Affiliations:** 1 Key Laboratory of Adolescent Health Assessment and Exercise Intervention of Ministry of Education, East China Normal University, Shanghai, 200241 China; 2 College of Physical Education and Health, East China Normal University, Shanghai, 200241 China; 3 College of Economics and Management, Shanghai Institute of Technology, Shanghai, 201418 China

**Keywords:** Population screening, Preventive medicine

## Abstract

To develop age- and sex-specific physical fitness reference standards and express the age- and sex-related differences using standardized effect sizes for Chinese children and adolescents. A total of 85,535 children and adolescents (48.7% girls) aged 7–18 years were recruited from six geographical divisions of China using a stratified randomized cluster sampling method. Seven physical fitness items including grip strength, standing long jump, 30-s sit-ups, sit and reach, 50-m dash, 20-s repeated straddling, and 20-m SRT were measured following a standardized procedure. Percentile curves for each physical fitness test were calculated using the LMS. Age- and sex-related differences were expressed as standardized effect sizes. We observed that the performance improved with age along with the analyzed percentiles in all tests. Boys had higher values compared to girls in all the physical fitness items except for sit and reach test, where girls showed better performance in all analyzed percentiles. Also, the sex differences increased with ages except sit and reach. There is a need for a differentiated approach in the physical education class in terms of adjustment of physical activity based on sex, level of fitness abilities in China.

## Introduction

Physical fitness is involved in the performance of the daily physical activity or physical exercise^1^. With multiple components, physical fitness includes cardiorespiratory fitness, musculoskeletal strength, endurance, flexibility, agility, balance^2^. Physical fitness has been considered as a predictor of morbidity and mortality for all causes^3^. A higher physical fitness level in children has been associated with more positive health-related outcomes^4^. While poor physical fitness is a risk factor for cardiovascular diseases^5^, diabetes mellitus^6^, and poor mental health^7^. Meanwhile, aspects of physical fitness in childhood are predictive of health outcomes later in life^8^.

Normative values of physical fitness, placing individuals and groups in percentiles and categories, can be used to help interpret an individual’s fitness test results by identifying how their results compare with the general population and helped with the diagnosis and prevention of diseases as well as in detecting sporting talents^9^. The developmental patterns of children and adolescents’ physical fitness have been well studied and extensively reviewed, such as USA^10^, Europe^11,12^, Spanish^13^, Canada^14^, and Australia^15^. We also developed 20 m-shuttle run test norms for Chinese children and adolescents^16^, while reference values are scarce for a comprehensive set of physical fitness tests.

Given the decline in physical fitness among Chinese children and adolescents^17^, the present study aimed: (1) to develop sex- and age-specific physical fitness reference standards; (2) to express sex- and age-related differences using standardized effect sizes, thus providing references for the improvement of Chinese children and adolescents.

## Method

### Participants

Data for this study were drawn from the “Preparation of New Evaluation Methods and Criteria for Physical Health of Children and Adolescents in China” (No. 11001-412221-15017). It was approved by the Human Experimental Ethics Committee of the East China Normal University (Approval No.: HR2016/12055) and was conducted in 2015–2016 by the Key Laboratory of Adolescent Health Assessment and Exercise Intervention of the Ministry of Education. A stratified randomized cluster sampling method was used to select participants from 27 provinces in six geographical divisions of China: East China (provinces including Shanghai, Shandong, Jiangsu, Zhejiang, Anhui, Jiangxi, Fujian), North China (provinces including Beijing, Neimenggu, Hebei, Shanxi), Central-South China (provinces including Henan, Hubei, Hunan, Guangdong, Guangxi, Hainan), Northwest China (provinces including Shanxi, Xinjiang, Gansu), Southwest China (provinces including Sichuan, Guizhou, Xizang, Yunnan), and Northeast China (provinces including Heilongjiang, Jilin, Liaoning). Public schools from urban and rural, decided by the administrative region of China, were selected in each province. Then classes were randomly selected from the selected schools. Subsequently, cluster students without physical and mental disabilities in the selected classes were recruited. The detailed sampling methods were also reported elsewhere^18^.

Finally, a total of 85,535 children and adolescents (48.7% girls) aged 7–18 years were involved in the present study. Participants included for each physical fitness test were presented in Table 1. Therein, 2.1% of the participants came from Lasa, Tibetan, which is 3500 m high than the sea level. Regarding nutritional status, BMI (kg/m^2^), calculated as body weight (kg) divided by height (m^2^), to define overweight and obesity and thinness according to the WHO standards and classifications^19^: thinness (< − 2 for BMI Z score), normal (≥ − 2 and ≤ 1 for BMI Z score), overweight (> 1 and ≤ 2for BMI Z score) and obesity (> 2 for BMI Z score). The prevalence of thinness, normal weight, overweight and obesity in the present study were1.9%, 68.9%, 12.8%, 16.4% for boys and 1.3%, 83.3%, 9.5%, 6.0% for girls, respectively. Before the investigation, verbal and written informed consent was obtained from both the students and their parents. All students’ names were digitally coded to avoid leaking their personal information.

**Table 1:** The averages and deviation of weight, height, and physical fitness tests by age by sex.

	N	Height	Weight	Grip strength	30-s sit-ups	Standing long jump	Sit and reach	20-m SRT	50-m dash	20-s repeated straddling
**Boys**										
7	4648	125.9 ± 6.8	26.5 ± 5.9	10.2 ± 2.9	14.3 ± 4.7	118.0 ± 20.8	27.8 ± 8.7	14.7 ± 6.4	11.3 ± 1.2	21.3 ± 7.0
8	3204	133.1 ± 7.1	30.8 ± 7.2	12.7 ± 3.2	16.5 ± 5.6	132.4 ± 20.2	30.9 ± 10.1	18.7 ± 8.1	10.4 ± 1.1	24.2 ± 7.4
9	3604	138.7 ± 7.4	34.1 ± 8.2	14.8 ± 3.5	18.2 ± 5.8	143.5 ± 21.6	33.3 ± 12.3	22.4 ± 10.0	9.9 ± 1.1	27.4 ± 7.2
10	3860	143.3 ± 8.0	37.7 ± 9.0	16.9 ± 4.0	19.2 ± 5.9	151.9 ± 21.5	33.6 ± 11.9	24.4 ± 10.8	9.6 ± 1.1	27.9 ± 7.7
11	3859	149.5 ± 8.2	42.0 ± 9.9	19.4 ± 5.1	20.2 ± 5.7	160.2 ± 22.2	33.8 ± 10.5	27.6 ± 11.6	9.3 ± 1.0	29.5 ± 7.8
12	3621	156.5 ± 9.4	47.6 ± 11.8	23.4 ± 6.6	21.5 ± 5.8	170.5 ± 25.3	34.6 ± 9.9	32.6 ± 13.9	8.9 ± 1.0	30.1 ± 8.8
13	3570	163.7 ± 9.0	53.4 ± 12.5	28.7 ± 7.7	22.7 ± 5.9	186.4 ± 26.0	36.4 ± 10.4	39.2 ± 16.3	8.4 ± 0.9	31.6 ± 8.8
14	3569	169.2 ± 7.5	57.1 ± 11.8	33.1 ± 8.0	25.0 ± 6.2	201.7 ± 27.9	38.7 ± 10.5	44.1 ± 17.6	8.0 ± 0.9	32.5 ± 8.4
15	3901	172.3 ± 6.8	61.0 ± 11.4	37.1 ± 8.8	25.7 ± 6.4	213.9 ± 26.7	38.6 ± 11.1	47.8 ± 19.0	7.7 ± 0.8	34.4 ± 8.3
16	3966	173.4 ± 6.5	63.0 ± 11.4	39.7 ± 8.4	25.7 ± 6.1	220.4 ± 26.5	38.5 ± 12.0	48.5 ± 19.7	7.6 ± 0.8	33.3 ± 9.9
17	3374	173.5 ± 6.3	63.6 ± 11.2	41.5 ± 8.1	25.5 ± 5.9	224.1 ± 26.0	39.2 ± 11.3	50.1 ± 19.8	7.5 ± 0.8	33.3 ± 10.4
18	2726	174.2 ± 6.2	64.5 ± 10.8	42.1 ± 8.1	25.1 ± 5.8	221.8 ± 26.1	38.4 ± 12.1	48.9 ± 19.8	7.5 ± 0.8	34.9 ± 9.8
Total	43,902	155.3 ± 18.6	47.8 ± 16.8	26.1 ± 13.1	21.5 ± 7.0	176.9 ± 43.9	35.1 ± 11.5	34.3 ± 19.6	8.9 ± 1.6	29.8 ± 9.4
**Girls**										
7	4060	124.6 ± 6.5	24.9 ± 5.0	9.4 ± 2.7	14.3 ± 4.9	113.0 ± 19.7	29.9 ± 9.0	14.4 ± 6.1	11.5 ± 1.2	21.6 ± 6.7
8	2978	131.5 ± 6.9	28.2 ± 5.8	11.5 ± 3.0	16.0 ± 5.6	126.5 ± 19.2	33.1 ± 10.1	17.6 ± 6.9	10.6 ± 1.1	24.0 ± 7.4
9	3490	137.4 ± 7.5	31.5 ± 6.8	13.5 ± 3.6	17.4 ± 5.3	138.0 ± 20.9	35.3 ± 12.0	21.1 ± 9.1	10.2 ± 1.1	27.0 ± 7.2
10	3458	143.7 ± 8.1	35.5 ± 8.0	15.7 ± 4.0	18.5 ± 5.4	145.9 ± 20.5	35.7 ± 12.0	22.9 ± 9.4	9.8 ± 1.0	27.6 ± 7.2
11	3428	150.3 ± 8.1	40.6 ± 8.7	18.3 ± 4.6	19.4 ± 5.4	152.1 ± 19.9	36.6 ± 11.2	25.4 ± 9.7	9.5 ± 0.9	28.8 ± 7.5
12	3279	155.6 ± 7.1	44.9 ± 8.9	20.9 ± 4.7	20.1 ± 5.2	157.2 ± 20.1	37.3 ± 10.2	28.1 ± 10.4	9.4 ± 0.9	29.0 ± 8.3
13	3150	159.6 ± 6.2	48.7 ± 8.5	23.1 ± 4.9	20.6 ± 5.3	162.8 ± 18.6	39.8 ± 11.0	32.0 ± 12.7	9.2 ± 0.9	29.8 ± 8.3
14	3294	161.2 ± 5.7	50.6 ± 8.0	23.7 ± 5.1	21.3 ± 5.3	165.6 ± 19.0	40.1 ± 10.4	32.2 ± 12.3	9.1 ± 0.9	29.6 ± 7.4
15	3705	161.8 ± 5.4	52.4 ± 7.6	25.0 ± 5.4	22.0 ± 5.7	170.4 ± 19.4	39.3 ± 10.6	32.4 ± 11.9	9.0 ± 0.8	30.3 ± 7.5
16	4010	162.1 ± 5.5	52.3 ± 7.2	26.2 ± 5.5	22.5 ± 5.8	170.6 ± 18.6	39.2 ± 11.8	31.0 ± 11.0	9.0 ± 0.9	29.0 ± 8.7
17	3623	161.8 ± 5.6	52.6 ± 7.2	26.6 ± 5.6	22.0 ± 5.7	170.7 ± 19.0	40.3 ± 10.9	31.9 ± 11.2	9.1 ± 0.9	29.5 ± 8.4
18	3158	162.0 ± 5.5	53.0 ± 7.0	26.7 ± 5.5	21.2 ± 5.4	168.6 ± 18.4	39.7 ± 11.1	30.4 ± 11.8	9.1 ± 0.9	29.3 ± 8.2
Total	41,633	150.9 ± 14.7	42.9 ± 12.6	20.0 ± 7.6	19.6 ± 6.0	153.3 ± 27.0	37.1 ± 11.3	26.5 ± 12.0	9.6 ± 1.3	27.9 ± 8.2

### Physical fitness measurement

All the measurements were carried out following relevant guidelines^20,21^ and regulations were conducted by trained staff. In each school, 1–2 professionals majored in human sport science and 4–5 trained and qualified physical education teachers were in charge of the physical fitness tests. To reduce measurement error, the measurement instruments were calibrated before use and each test was completed at a fixed time of the day to reduce data deviation caused by different test times. Physical fitness items included grip strength (reflecting upper-body strength), standing long jump (reflecting lower limb strength), 30-s sit-ups (reflecting abdominal strength), sit and reach (reflecting flexibility), 50-m dash (reflecting speed), 20-s repeated straddling (reflecting agility), and 20-m shuttle run test (20-m SRT, reflecting cardiorespiratory fitness).

#### Grip strength

Participants were requested to stand upright with feet shoulder-width apart and elbow fully extended during the assessment. Then they were instructed to squeeze the grip with full force and continuously for at least two seconds twice. The larger value was recorded.

#### Standing long jump

The participant was instructed to stand behind the starting line (but as close to it as possible) to prepare for the upcoming standing long jump. Each participant was instructed to push off vigorously and jump horizontally as far as possible, taking off and landing with the feet together and to stay upright. The distance from the starting line to the heel of the foot closest to the start line was recorded. The test was repeated twice and the best score was retained in centimeters.

#### 30-s sit-ups

The participants were requested to lay relaxed on the cushion, with feet pressed by an assistant and hands crossed over the chest to prepare the test of 30 s sit-ups. When heard the starting signal, the participant repeatedly sat up and touched his knee with the forehead, then lay down quickly. The times of the forehead touching the knee within 30 s is recorded as the result.

#### Sit and reach

The participant sat on a mat with shoes removed, with both legs shoulder-width apart and fully extended, heels on the pad of the instrument. The height of the guide rail was adjusted to keep the participant’s toes even with the lower edge of the marker. The participant was then instructed to slowly reach forward and push the marker forward with the middle fingertips of both hands as far as possible on the scale. Two trials were completed, and the greater distance was recorded as the result of the sit and reach test.

#### 50-m dash

The result of the 50-m dash was the time taken to run 50 m from the starting line. The participants were instructed to run toward the finish line as fast as they could immediately on hearing the starting signal. The result was recorded to the nearest 0.1 s.

#### 20-s repeated straddling

There were three parallel lines 100 cm apart on the ground. The participants stood across the central line and moved horizontally to the right line then back to the central, left, central, and so on when heard the starting signal. Jumping is prohibited. The number of straddles in 20 s was recorded as a result.

#### 20-m SRT

20-m SRT involves continuous running back and forth between two parallel lines 20-m apart in time to audio signals. It comprises several stages (also called levels), each lasting about one minute, with each stage comprising many 20-m laps (also called shuttles). At each stage, the required running speed increases, until the child can no longer run the 20-m distance in time with the audio signal (on 2 consecutive occasions) or when the child stops due to volitional fatigue. The last lap completed was recorded as the result.

### Statistical analysis

All statistical analyses were performed using the LMS Chart maker Pro version 2.43 (Institute of Child Health, Lon-don) and SPSS version 25.0 (IBM, Armonk, NY, USA). The level of statistical significance was set at 0.05. Percentile curves for each physical fitness test were calculated using the LMS, which summarizes the changing distribution in reference centile curves, representing skewness (L, expressed as a Box-Cox power transformation), median (M), and coefficient of variation (S). Smooth centile curves were fitted to obtain the sex- and age-specific norms for Chinese children and youth and the effective degrees of freedom in the present study were 2 (L curve), 4 (M curve), and 2 (S curve) for both boys and girls. At last, the age- and sex-specific percentile values were calculated for each physical fitness test.

Age- and sex-related differences in means were expressed as standardized effect sizes for each fitness test. In the age-related analysis, taking the mean of each test of 7 years boys and girls as reference respectively, standardized effect sizes of 8–18 years old children and adolescents were calculated. Similarly, in sex-related analysis, taking the mean of each test of 7–18 years girls as reference respectively, standardized effect sizes of 7–18 years old boys were obtained. Positive effect sizes indicated that mean fitness test performances for older children (in age-related analysis) or boys (in sex-related analysis) were higher than those for 7 years old children or girls. Effect sizes of 0.2, 0.5, and 0.8 were used as thresholds for small, moderate, and large^22^.

## Result

**Figure 1:**
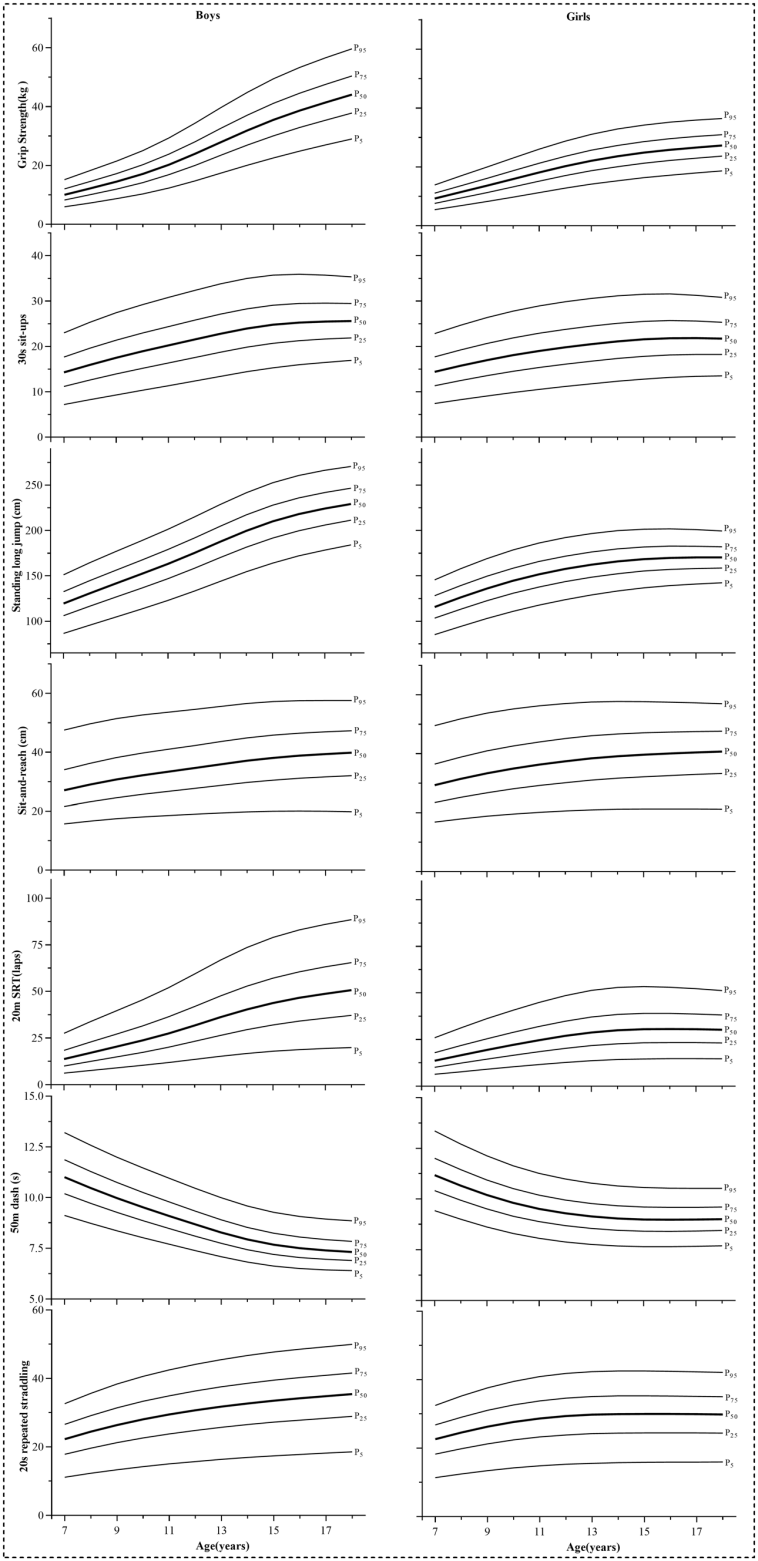
Smoothed centile curves of seven physical fitness tests for Chinese children and adolescents.

Table 1 showed the averages and deviation of weight, height, and physical tests by age and sex. Table 2 showed the sex‑ and age‑specific percentile values (5th, 15th, 25th, 35th, 45th, 50th, 55th, 65th, 75th, 85th, and 95th percentiles) for each physical fitness test. Figure 1 showed the percentile curves for the 5th, 25th 50th, 75th, and 95th percentiles for all the physical fitness measures across different age and sex groups. In general, the performance improved with age along with the analyzed percentiles for most tests. For example, from 7 to 18 years old, the score of standing long jump increased by 91.8% for boys and 47.0% for girls at P50 (Table 2).

**Table 2:** Reference standards of the seven physical fitness tests by age and sex for Chinese children and adolescents.

	Boys										
	P5	P15	P25	P35	P45	P50	P55	P65	P75	P85	P95
**Grip strength**											
7	5.9	7.3	8.2	8.9	9.6	10.0	10.4	11.1	12.0	13.1	15.1
8	7.2	8.9	10.0	10.9	11.8	12.2	12.6	13.5	14.6	15.9	18.3
9	8.6	10.7	11.9	13.0	14.0	14.5	15.0	16.0	17.2	18.7	21.4
10	10.3	12.7	14.1	15.4	16.5	17.1	17.6	18.8	20.2	21.9	25.0
11	12.3	15.1	16.8	18.2	19.6	20.2	20.9	22.2	23.8	25.8	29.3
12	14.7	18.0	20.0	21.6	23.2	23.9	24.7	26.3	28.0	30.3	34.3
13	17.4	21.1	23.4	25.3	27.1	27.9	28.8	30.6	32.6	35.2	39.7
14	20.0	24.3	26.9	29.0	30.9	31.9	32.8	34.8	37.1	39.9	44.8
15	22.5	27.2	30.0	32.3	34.5	35.5	36.5	38.7	41.1	44.2	49.4
16	24.8	29.8	32.9	35.3	37.5	38.6	39.7	42.0	44.5	47.8	53.3
17	26.9	32.2	35.4	38.0	40.3	41.4	42.6	44.9	47.6	50.9	56.6
18	29.0	34.5	37.8	40.5	42.9	44.0	45.2	47.6	50.4	53.8	59.6
**30-s sit-ups**											
7	7.2	9.6	11.2	12.5	13.7	14.3	14.9	16.2	17.7	19.6	23.0
8	8.3	10.9	12.6	14.0	15.3	15.9	16.6	18.0	19.6	21.7	25.3
9	9.3	12.1	13.9	15.4	16.8	17.5	18.2	19.7	21.4	23.6	27.4
10	10.3	13.3	15.2	16.7	18.2	18.9	19.6	21.2	22.9	25.2	29.2
11	11.3	14.4	16.3	18.0	19.5	20.2	21.0	22.6	24.4	26.7	30.8
12	12.3	15.5	17.5	19.2	20.8	21.5	22.3	23.9	25.8	28.2	32.3
13	13.4	16.7	18.7	20.4	22.0	22.8	23.6	25.2	27.1	29.5	33.8
14	14.4	17.7	19.8	21.6	23.1	23.9	24.7	26.4	28.3	30.7	35.0
15	15.3	18.6	20.7	22.4	24.0	24.8	25.5	27.2	29.1	31.5	35.7
16	16.0	19.2	21.3	22.9	24.5	25.3	26.0	27.6	29.5	31.8	35.9
17	16.5	19.6	21.6	23.3	24.7	25.5	26.2	27.8	29.5	31.8	35.7
18	16.9	20.0	21.9	23.4	24.9	25.6	26.3	27.8	29.4	31.6	35.3
**Standing long jump**											
7	86.6	98.9	106.2	111.9	117.0	119.5	122.0	127.0	132.6	139.6	151.2
8	95.6	108.8	116.5	122.6	128.1	130.7	133.3	138.7	144.6	152.0	164.3
9	104.6	118.5	126.6	133.1	138.8	141.6	144.3	150.0	156.2	164.0	176.9
10	113.5	128.1	136.6	143.3	149.3	152.2	155.1	161.0	167.5	175.6	189.0
11	122.9	138.1	147.0	154.0	160.2	163.2	166.2	172.3	179.1	187.5	201.4
12	133.1	149.0	158.2	165.5	172.0	175.1	178.2	184.6	191.6	200.3	214.7
13	143.9	160.5	170.1	177.7	184.4	187.6	190.8	197.4	204.7	213.7	228.6
14	154.5	171.6	181.6	189.4	196.3	199.6	202.9	209.7	217.2	226.4	241.7
15	164.1	181.5	191.6	199.5	206.6	209.9	213.3	220.2	227.8	237.1	252.6
16	172.0	189.5	199.6	207.6	214.6	218.0	221.4	228.3	235.8	245.2	260.6
17	178.4	195.9	205.9	213.8	220.8	224.2	227.5	234.3	241.8	251.0	266.2
18	184.2	201.4	211.3	219.1	225.9	229.2	232.5	239.2	246.5	255.6	270.5
**Sit and reach**											
7	15.6	19.1	21.5	23.7	25.9	27.0	28.2	30.8	34.0	38.5	47.5
8	16.6	20.4	23.1	25.5	27.8	29.0	30.2	32.9	36.2	40.7	49.6
9	17.4	21.6	24.5	27.0	29.5	30.7	32.0	34.8	38.1	42.7	51.4
10	18.0	22.6	25.7	28.3	30.8	32.1	33.5	36.3	39.7	44.2	52.6
11	18.5	23.4	26.7	29.4	32.0	33.4	34.7	37.6	41.0	45.4	53.6
12	18.9	24.2	27.6	30.5	33.2	34.6	36.0	38.9	42.2	46.6	54.5
13	19.4	25.1	28.7	31.7	34.5	35.8	37.2	40.2	43.6	47.9	55.6
14	19.8	25.9	29.7	32.8	35.6	37.1	38.5	41.4	44.8	49.1	56.5
15	20.0	26.5	30.5	33.7	36.6	38.0	39.5	42.4	45.8	50.0	57.2
16	20.0	27.0	31.1	34.4	37.3	38.8	40.2	43.2	46.5	50.6	57.5
17	19.9	27.3	31.6	34.9	37.9	39.3	40.8	43.7	46.9	50.9	57.6
18	19.8	27.6	32.0	35.4	38.4	39.8	41.2	44.1	47.3	51.2	57.6
**20-m SRT**											
7	6	8	10	11	13	14	14	16	18	21	28
8	8	10	12	14	16	17	18	20	23	26	34
9	9	12	15	17	19	20	21	24	27	31	40
10	10	14	17	20	22	24	25	28	31	36	45
11	12	17	20	23	26	27	29	32	36	42	52
12	14	19	23	27	30	32	34	37	42	48	59
13	15	22	26	30	34	36	38	43	48	54	67
14	17	24	29	34	38	40	43	47	53	60	74
15	18	26	32	37	41	44	46	51	57	65	79
16	19	28	34	39	44	47	49	54	61	69	83
17	19	29	36	41	46	49	51	57	63	71	86
18	20	30	37	43	48	51	53	59	65	74	88
**50-m dash**											
7	9.1	9.8	10.2	10.5	10.8	11.0	11.2	11.5	11.9	12.3	13.2
8	8.7	9.3	9.7	10.0	10.3	10.5	10.6	10.9	11.3	11.8	12.6
9	8.4	8.9	9.3	9.6	9.8	10.0	10.1	10.4	10.7	11.2	12.0
10	8.0	8.5	8.9	9.1	9.4	9.5	9.6	9.9	10.2	10.7	11.5
11	7.7	8.2	8.5	8.7	9.0	9.1	9.2	9.5	9.8	10.2	11.0
12	7.4	7.8	8.1	8.3	8.6	8.7	8.8	9.0	9.3	9.7	10.5
13	7.1	7.5	7.7	8.0	8.2	8.3	8.4	8.6	8.9	9.3	10.0
14	6.8	7.2	7.4	7.6	7.8	7.9	8.0	8.3	8.5	8.9	9.6
15	6.6	7.0	7.2	7.4	7.6	7.7	7.8	8.0	8.2	8.6	9.3
16	6.5	6.8	7.0	7.2	7.4	7.5	7.6	7.8	8.0	8.4	9.1
17	6.4	6.7	7.0	7.1	7.3	7.4	7.5	7.7	7.9	8.3	8.9
18	6.4	6.7	6.9	7.1	7.2	7.3	7.4	7.6	7.8	8.2	8.8
**20-s repeated straddling**											
7	11.1	15.4	17.8	19.8	21.5	22.3	23.1	24.8	26.6	28.9	32.6
8	12.3	16.9	19.6	21.7	23.5	24.4	25.3	27.1	29.1	31.6	35.6
9	13.3	18.3	21.2	23.5	25.4	26.4	27.3	29.3	31.4	34.0	38.4
10	14.2	19.6	22.6	25.0	27.1	28.1	29.0	31.1	33.3	36.1	40.6
11	15.0	20.6	23.8	26.2	28.4	29.5	30.5	32.6	34.9	37.8	42.5
12	15.7	21.5	24.8	27.4	29.6	30.7	31.7	33.9	36.3	39.3	44.1
13	16.3	22.3	25.7	28.3	30.6	31.8	32.8	35.1	37.5	40.6	45.5
14	16.9	23.0	26.5	29.2	31.6	32.7	33.8	36.1	38.6	41.7	46.7
15	17.4	23.7	27.2	30.0	32.4	33.5	34.6	37.0	39.5	42.6	47.7
16	17.8	24.2	27.8	30.6	33.0	34.2	35.3	37.7	40.2	43.4	48.5
17	18.2	24.7	28.4	31.2	33.6	34.8	36.0	38.3	40.9	44.1	49.3
18	18.6	25.2	28.9	31.8	34.2	35.4	36.6	39.0	41.6	44.8	50.0

	Girls										
	P5	P15	P25	P35	P45	P50	P55	P65	P75	P85	P95
**Grip strength**											
7	5.4	6.7	7.6	8.3	8.9	9.2	9.5	10.2	11.0	12.0	13.8
8	6.8	8.4	9.4	10.2	11.0	11.4	11.8	12.6	13.5	14.7	16.9
9	8.2	10.1	11.2	12.2	13.1	13.6	14.0	15.0	16.1	17.5	19.9
10	9.7	11.8	13.2	14.3	15.3	15.8	16.3	17.4	18.7	20.2	23.0
11	11.2	13.6	15.2	16.4	17.5	18.1	18.7	19.9	21.2	23.0	26.0
12	12.7	15.4	17.0	18.4	19.6	20.2	20.8	22.1	23.6	25.5	28.8
13	14.1	16.9	18.7	20.1	21.4	22.1	22.7	24.1	25.6	27.6	31.1
14	15.3	18.2	20.1	21.5	22.9	23.6	24.3	25.7	27.3	29.3	32.9
15	16.3	19.3	21.2	22.7	24.1	24.8	25.5	26.9	28.6	30.6	34.2
16	17.2	20.2	22.1	23.7	25.1	25.8	26.5	27.9	29.5	31.6	35.2
17	17.9	21.0	22.9	24.5	25.9	26.6	27.2	28.7	30.3	32.4	35.9
18	18.6	21.7	23.6	25.2	26.6	27.2	27.9	29.3	30.9	33.0	36.4
**30-s sit-ups**											
7	7.4	9.8	11.3	12.6	13.8	14.4	15.0	16.2	17.7	19.5	22.8
8	8.2	10.8	12.5	13.8	15.1	15.7	16.4	17.7	19.2	21.2	24.7
9	9.1	11.8	13.5	15.0	16.3	17.0	17.6	19.0	20.7	22.7	26.4
10	9.8	12.7	14.5	16.0	17.4	18.1	18.8	20.2	21.9	24.0	27.8
11	10.5	13.5	15.3	16.9	18.3	19.0	19.7	21.2	22.9	25.1	28.9
12	11.2	14.2	16.1	17.6	19.1	19.8	20.5	22.0	23.8	26.0	29.8
13	11.8	14.8	16.7	18.3	19.8	20.5	21.2	22.8	24.5	26.7	30.6
14	12.3	15.4	17.3	18.9	20.4	21.1	21.8	23.4	25.1	27.3	31.1
15	12.8	15.9	17.8	19.4	20.8	21.6	22.3	23.8	25.5	27.7	31.5
16	13.2	16.2	18.1	19.7	21.1	21.8	22.5	24.0	25.7	27.8	31.6
17	13.4	16.4	18.2	19.7	21.1	21.8	22.5	24.0	25.6	27.7	31.3
18	13.5	16.4	18.2	19.7	21.0	21.7	22.4	23.8	25.3	27.3	30.8
**Standing long jump**											
7	85.2	96.6	103.4	108.7	113.5	115.8	118.1	122.8	128.0	134.6	145.5
8	94.3	106.2	113.2	118.8	123.8	126.3	128.7	133.7	139.2	146.2	157.8
9	103.0	115.2	122.5	128.3	133.5	136.1	138.6	143.8	149.6	156.9	169.0
10	110.9	123.3	130.7	136.7	142.0	144.6	147.2	152.5	158.5	166.0	178.6
11	117.8	130.3	137.7	143.7	149.1	151.7	154.4	159.8	165.8	173.4	186.2
12	123.8	136.2	143.6	149.6	155.0	157.6	160.2	165.6	171.7	179.3	192.2
13	128.9	141.1	148.5	154.4	159.7	162.3	164.9	170.3	176.3	183.8	196.6
14	133.3	145.2	152.4	158.2	163.4	165.9	168.5	173.7	179.6	187.1	199.7
15	136.7	148.3	155.3	160.9	166.0	168.4	170.9	176.1	181.8	189.1	201.4
16	139.3	150.4	157.1	162.5	167.4	169.8	172.2	177.2	182.7	189.8	201.7
17	141.1	151.7	158.1	163.3	168.0	170.3	172.5	177.3	182.6	189.4	200.9
18	142.4	152.5	158.6	163.6	168.0	170.2	172.4	176.9	182.0	188.5	199.5
**Sit and reach**											
7	16.8	20.7	23.4	25.7	28.1	29.2	30.5	33.2	36.4	40.9	49.5
8	17.8	22.2	25.1	27.7	30.1	31.4	32.7	35.5	38.8	43.3	51.8
9	18.8	23.5	26.7	29.4	32.0	33.3	34.6	37.5	40.9	45.4	53.8
10	19.5	24.6	28.0	30.8	33.5	34.9	36.3	39.2	42.6	47.1	55.2
11	20.1	25.6	29.1	32.1	34.8	36.2	37.6	40.6	44.0	48.4	56.2
12	20.6	26.4	30.1	33.2	36.0	37.4	38.8	41.7	45.1	49.4	57.0
13	20.9	27.1	31.0	34.1	37.0	38.4	39.8	42.7	46.1	50.3	57.6
14	21.1	27.7	31.6	34.8	37.7	39.1	40.5	43.4	46.7	50.8	57.7
15	21.2	28.1	32.1	35.3	38.2	39.6	41.0	43.9	47.0	51.0	57.7
16	21.2	28.4	32.5	35.8	38.7	40.0	41.4	44.2	47.3	51.1	57.5
17	21.2	28.7	32.9	36.2	39.0	40.4	41.7	44.5	47.5	51.2	57.2
18	21.2	29.0	33.3	36.5	39.3	40.7	42.0	44.7	47.6	51.2	56.9
**20-m SRT**											
7	6	9	10	12	13	14	14	16	18	21	26
8	8	10	12	14	16	17	17	19	22	25	31
9	9	12	14	16	18	19	20	23	25	29	36
10	10	14	17	19	21	22	23	26	29	33	41
11	12	16	18	21	23	25	26	29	32	36	45
12	13	17	20	23	26	27	28	31	35	40	49
13	14	18	22	25	27	29	30	33	37	42	51
14	14	19	23	26	29	30	31	35	38	44	53
15	15	20	23	26	29	31	32	35	39	44	53
16	15	20	23	26	29	31	32	35	39	44	53
17	15	20	23	26	29	30	32	35	39	43	52
18	15	20	23	26	29	30	32	35	38	43	51
**50-m dash**											
7	9.4	10.0	10.4	10.7	11.0	11.2	11.3	11.6	12.0	12.5	13.4
8	9.0	9.6	9.9	10.2	10.5	10.7	10.8	11.1	11.4	11.9	12.7
9	8.6	9.2	9.5	9.8	10.1	10.2	10.3	10.6	10.9	11.4	12.1
10	8.3	8.8	9.2	9.4	9.7	9.8	9.9	10.2	10.5	10.9	11.6
11	8.1	8.6	8.9	9.1	9.4	9.5	9.6	9.9	10.2	10.6	11.3
12	7.9	8.4	8.7	8.9	9.2	9.3	9.4	9.7	9.9	10.3	11.0
13	7.8	8.2	8.5	8.8	9.0	9.1	9.3	9.5	9.8	10.1	10.8
14	7.7	8.2	8.5	8.7	8.9	9.0	9.2	9.4	9.7	10.0	10.6
15	7.6	8.1	8.4	8.7	8.9	9.0	9.1	9.3	9.6	10.0	10.6
16	7.6	8.1	8.4	8.6	8.9	9.0	9.1	9.3	9.6	9.9	10.5
17	7.7	8.1	8.4	8.7	8.9	9.0	9.1	9.3	9.6	9.9	10.5
18	7.7	8.2	8.4	8.7	8.9	9.0	9.1	9.3	9.6	9.9	10.5
**20-s repeated straddling**											
7	11.4	15.8	18.2	20.1	21.8	22.6	23.4	25.0	26.8	28.9	32.5
8	12.5	17.2	19.8	21.9	23.7	24.5	25.4	27.1	29.0	31.4	35.2
9	13.4	18.4	21.3	23.4	25.4	26.3	27.2	29.0	31.0	33.5	37.6
10	14.2	19.5	22.4	24.7	26.7	27.6	28.6	30.5	32.6	35.2	39.5
11	14.8	20.2	23.3	25.6	27.7	28.7	29.6	31.6	33.8	36.5	40.9
12	15.3	20.8	23.9	26.2	28.4	29.4	30.4	32.4	34.6	37.3	41.8
13	15.6	21.1	24.2	26.6	28.8	29.8	30.8	32.8	35.1	37.8	42.3
14	15.7	21.3	24.4	26.8	28.9	30.0	31.0	33.0	35.3	38.0	42.5
15	15.8	21.4	24.5	26.9	29.0	30.0	31.0	33.0	35.3	38.0	42.5
16	15.9	21.4	24.5	26.9	29.0	30.0	31.0	33.0	35.2	38.0	42.4
17	15.9	21.4	24.4	26.8	28.9	29.9	30.9	32.9	35.1	37.8	42.3
18	15.9	21.4	24.4	26.8	28.8	29.8	30.8	32.8	35.0	37.7	42.1

**Figure 2:**
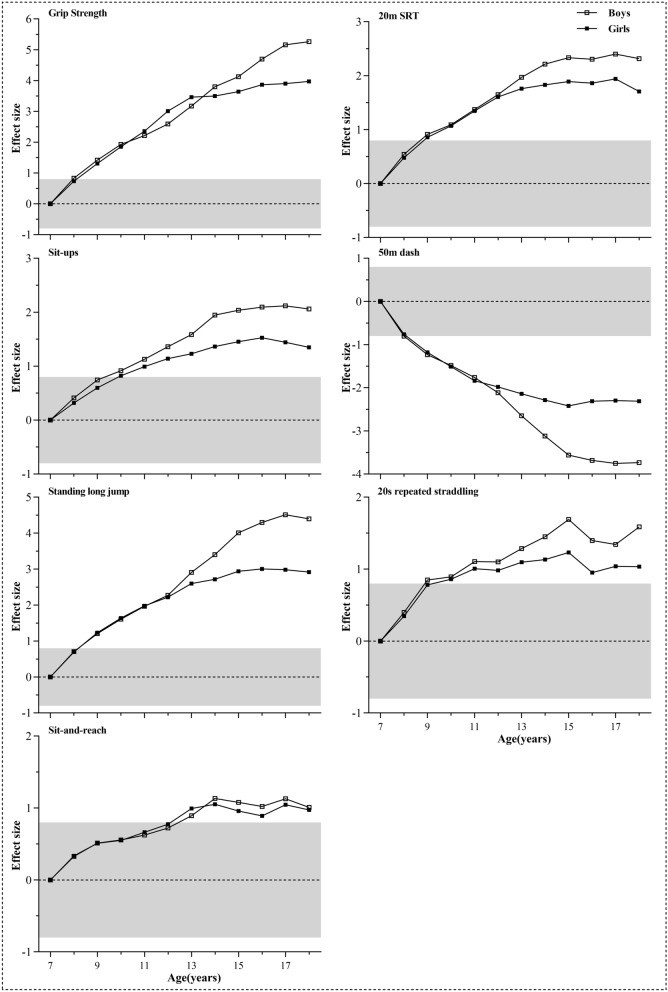
Age-related difference in each physical fitness test ((**A**) grip strength, (**B**) 20-m SRT, (**C**) 30-s sit-ups, (**D**) 50-m dash, (**E**) standing long jump, (**F**) 20-s repeated straddling, (**G**) sit and reach) expressed as standardized effect sizes (anchored to age 7 years = 0). The limits of the grey zone represent the threshold for a large standardized difference (i.e., 0.8 or − 0.8). Positive effect sizes indicated that mean fitness test performances for older children and adolescents were higher than those for 7 years old children.

Age-related differences for each test were shown in Fig. 2. It can be observed large differences in high age groups such as over 8 years for grip strength and standing long jump. The largest rate of increase occurring in teenage years especially for muscular fitness tests. Boys had considerably better performances than girls in grip strength, standing long jump, and 50-m dash across all ages and along with the analyzed percentiles. Taken grip strength as an example, boys outperformed girls by 6.6% at 9 years old and 61.7% at 18 years old at 50th percentiles. While the advantages of boys at 7 years old slightly declined since girls at this age performed better in 30-s sit-ups below P60, 20-m SRT at P10 and 20-s repeated straddling at most of the percentiles. When it comes to sit and reach, girls had better values than boys with the biggest difference (8.7%) at 10 years old in P50.

**Figure 3:**
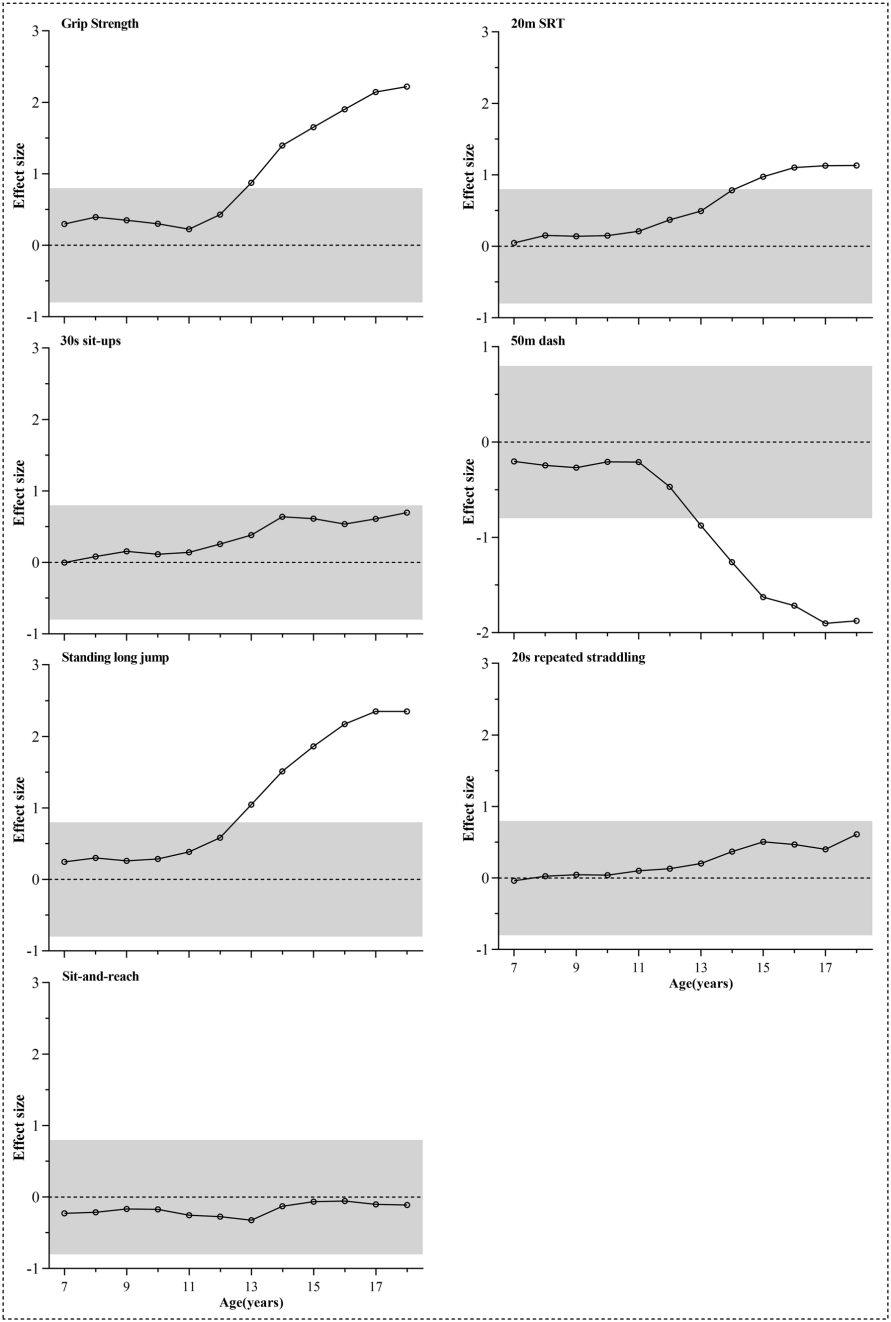
Sex-related difference in each physical fitness test ((**A**) grip strength, (**B**) 20-m SRT, (**C**) 30-s sit-ups, (**D**) 50-m dash, (**E**) standing long jump, (**F**) 20-s repeated straddling, (**G**) sit and reach) expressed as standardized effect sizes (anchored to girls = 0). The limits of the grey zone represent the threshold for a large standardized difference (i.e., 0.8 or − 0.8). Positive effect sizes indicated that mean fitness test performances for boys were higher than those for girls.

Sex-related differences for each test were shown in Fig. 3. Large differences can be observed for grip strength, standing long jump, 50-m dash, and 20-m SRT over 13 or 14 years old, from which the increased sex differences with age can also be obtained.

## Discussion

The present study used nationally representative data on physical fitness to develop sex- and age-specific norms for Chinese children and adolescents, which can be used as benchmark values for health and fitness screening and surveillance. We observed that the performance improved with age along with the analyzed percentiles in all tests. Boys had higher values compared to girls in all the physical fitness items except for sit and reach test, where girls showed better performance in all analyzed percentiles. Also, the sex differences increased with ages except sit and reach.

Comparing the international studies with the results obtained in our study, it can be concluded that, taking boys aged 11 years at P50 as an example, cardiorespiratory fitness resulted similar for China (6.0 stages/minutes) and Spanish (5.8 stages/minutes, for 16–17 years boys)^23^, but worse than Australian (8 stages/minutes)^15^. Regarding lower limb muscle strength, Chinese girls aged 11 years at P50 had better performances (151.7 cm) of French (127 cm)^24^, Macedonian (127.9 cm)^25^, and Australian (140 cm)^15^. Finally, Chinese children and youth underperformed in speed capability than their Australian counterparts (9.0 s vs 8.6 s)^15^.

The results from this study generally align with findings from previous research, such as for European children^11^, Australian children^15^. This study’s findings for the increasing physical fitness with age support previous Canadian and French studies^9,26^. We found that for boys and girls, the performance in physical fitness tests increased with increasing age especially for grip strength, in which P50 increased averagely by 3.1 kg as age increased 1 year for boys, and 1.64 kg for girls. The factors of this age-difference may be included motivation, concentration, the degree of motor skills, physical activity, and body composition^27^.

Another finding of our study is that physical fitness levels were better in boys than girls, except for flexibility (sit and reach test), where girls have achieved better results. This finding agrees with the results previously reported in children and adolescents^11,28^. Moreover, it was reported that sex differences in physical fitness (i.e. cardiorespiratory fitness, muscular strength, and speed-agility) are detectable as early as preschool age^29^. Distinct development, growth, and maturation of boys and girls undoubtedly contribute to these differences, while the sex differences in physical fitness performance in our study might also be related to the effects of genetics, anatomy, physiology, behavior, and social and physical environments^30,31^. Carlos et al. investigated the magnitude of sex differences in physical fitness and suggested that greater sex differences in the explosive strength of upper and lower limbs, and smaller in the abdominal and upper limbs muscular endurance and trunk extensor strength and flexibility, balance, and speed^32^. Recent studies have identified that boys outperformance in cardiorespiratory fitness and muscular strength because they are more physically active and have a higher fat-free mass^33^. Regarding the flexibility, some of the factors presented for better performance of girls are that girls have greater passive dorsiflexion angle, while boys have a higher muscle volume and dynamic property of tendon tissues^34^.

We also observed sex differences also increased with age. The P50 differences of cardiorespiratory fitness between boys and girls increased from 1 lap in 9 years old to 21 laps in 18 years old. Consistent with this study’s findings, other studies in children and adolescents showed a similar sex-differences trend in P50, which was + 38 laps for boys in 18-year-old adolescents^11^. The higher age-related sex differences in adolescents compared to children might be explained by more pronounced physiological changes caused by pubertal development^30,35^. Sex and age-related differences reflect the complex and interconnected effects of genetics, anatomy, physiology, behavior, social, and physical environments^14,36^.

This study has several strengths, including the large sample of children and adolescents from across China with sex-specific information, and the harmonization and standardization of assessment of physical fitness. Despite these strengths, this study is not without limitations. The main limitation of the study is the cross-sectional design, which prevents the examination of inter- and intra-individual differences, resulting in the need for a longitudinal study with repeated measurements. Besides, differences during the maturation can’t be excluded since we didn’t take the physical growth or biological maturity into account.

## Conclusion

The present study produced nationally representative normative‑referenced percentile values for seven physical fitness tests. All these norms suggested sex-based differences in physical fitness and older children performed better than younger children. Thus, there is a need for a differentiated approach in the physical education class in terms of adjustment of physical activity based on sex, age, and level of fitness abilities.

## Data Availability

The datasets generated during and/or analyzed during the current study are available from the corresponding author on reasonable request.
